# A functional mutation at position -155 in porcine APOE promoter affects gene expression

**DOI:** 10.1186/1471-2156-12-40

**Published:** 2011-05-09

**Authors:** Shixin Li, Hao Zhang, Ping Gao, Zanmou Chen, Chong Wang, Jiaqi Li

**Affiliations:** 1 Guangdong Provincial Key Lab of Agroanimal Genomics and Molecular Breeding, College of Animal Science, South China Agricultural University, Guangzhou 510642, Guangdong, PR China; 2 Department of Animal Genetics, Breeding and Reproduction, College of Animal Science, South China Agricultural University, Guangzhou 510642, Guangdong, PR China; 3 Laboratory of Animal Physiology and Biochemistry, College of Animal Science, South China Agricultural University, Guangzhou 510642, Guangdong, PR China; 4 College of Life Sciences, Zhongkai University of Agriculture and Engineering, Guangzhou 510225, Guangdong, PR China

## Abstract

**Background:**

Apolipoprotein E, a component of the plasma lipoproteins, plays an important role in the transport and metabolism of cholesterol and other lipids. Three single nucleotide polymorphisms (SNPs) -491A>T, -219T>G and +113G>C in the regulatory region of human apolipoprotein E gene (*APOE*) change the promoter activity and are associated with a wide variety of disorders including Alzheimer disease (AD). Functional SNPs in porcine *APOE *gene 5' regulatory region have not been explored.

**Results:**

We examined SNPs within this region (from -831 to +855), and the analysis revealed that the T>A SNP at position -155 among these three polymorphism sites (-440, -155, +501) was found to exert a marked influence on the transcription of the porcine *APOE *gene. Electrophoretic mobility shift assays showed that the binding affinity of oligonucletides containing the -155A to transcription factor(s) was stronger than that of the -155T. Transient transfection assays and quantitative real-time PCR results revealed that the -155T>A variant enhanced the activity of the *APOE *promoter and was associated with increased *APOE *mRNA levels *in vivo*.

**Conclusions:**

These data suggest that the -155T>A mutation in the promoter region of the porcine *APOE *gene is an important functional variant. The results provided new insights into aspects of pig genetics and might also facilitate the application of pigs in biomedical studies addressing important human diseases.

## Background

Apolipoprotein E (apoE = protein; *APOE *= gene) is a component of lipoproteins, and thereby regulates lipoprotein metabolism; apoE also plays a key role in maintaining neuronal integrity [1-4]. Utermann et al. [5] were the first to identify three isoforms of human apoE, named E2, E3, and E4. The allele encoding apoE4 is a risk factor for atherosclerosis [6,7], and AD [1,8,9]. In addition, humans with E4 allele responded to a lipid-lowering therapy poorly whereas those with E2 allele sensitively [10]. There are four single nucleotide polymorphisms (SNPs) at -491, -427, -219 and +113 in human *APOE *promoter [11-13]. The base substitutions at -491A>T and -219T>G were found to alter promoter activity and transcription factor binding affinity [12,14,15]. The -491SNP and -219SNP were related to different plasma apoE [16], LDL and cholesterol concentrations [17], and the risks of atherosclerosis [16] and AD [14,15]. Furthermore, +113SNP modulated lipid, lipoprotein concentrations and aortic atherosclerosis [11,13]. However, there were inconsistent reports about *APOE *polymorphisms and coronary heart disease [18] or AD [19-21].

In pigs, *APOE *has been mapped to chromosome 6 [22,23]. Porcine *APOE *is 4267 nucleotides in length, comprising of four exons and three introns, and a (CG)_13 _microsatellite located within intron 3 [24]. Additionally, Brzozoeska et al. [25] studied the cDNA sequence of porcine *APOE*, and Kurył [23] described three isoforms of porcine apoE. Fan et al. [26] recently demonstrated that a SNP within intron 2 of porcine *APOE *was associated with body conformation traits. However, the functional SNPs in the 5' regulatory region of porcine *APOE *remain unclear.

To identify the functional SNPs in the 5' regulatory region of porcine *APOE*, mRNA expression levels and promoter activities associated with different genotypes were analyzed with quantitative RT-PCR (qRT-PCR) and transient transfection assays respectively, potential *cis*-acting elements surrounding the SNP were examined with electrophoretic mobility shift assays (EMSAs). Our results indicate that the -155 SNP modulates the expression level of porcine *APOE*.

## Results

### Screening for SNPs in the APOE 5' regulatory region

Three overlapping fragments from -831 to +855 (1686 bp) were amplified. Three SNPs were identified in this region: -155T>A, -440G>A, and +501A>T.

### Genotype frequencies of -155T>A SNP

The genotypic frequencies of -155SNP were listed in Table 1. The frequencies were highly significant among different genotypes within the breeds (*P *< 0.01), but not significantly different (*P *> 0.05) between the breeds.

**Table 1:** Genotype frequencies of the porcine *APOE *-155 SNP in different populations.

**Breed**	**N**	**Genotype**		
		
		**TT**	**TA**	**AA**
Dahuabai	58	20(0.34)	30(0.52)	8(0.14)
Lantang	50	22(.44)	24(0.48)	4(0.08)
Yorkshire	51	26(0.51)	19(0.37)	6(0.12)
Landrace	50	24(0.48)	25(0.50)	1(0.02)

N, Number of animals.

### Alternations of predicted transcription factor binding sites surrounding porcine *APOE *SNPs

The three polymorphic sites were examined with MatInspector http://www.genomatix.de[27]. Results revealed that the mutation -155 T>A lost the transcription factor sites for GAGA, KLF6, PURα, KKLF, cKROX and MAZ, and gained sites for BKLF and CTCF (additional file 1), while the other two (-440G>A, and +501A>T) did not show changes (data not shown).

### Alignment of porcine and human *APOE *promoter sequences

**Figure 1:**
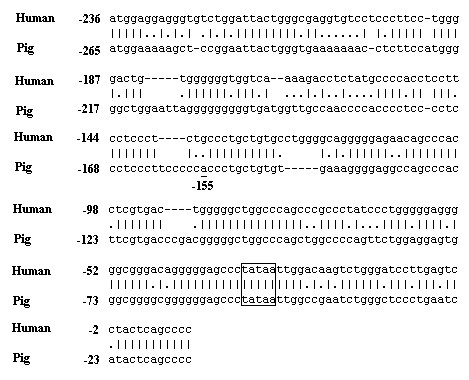
**Homology of human *APOE *and porcine *APOE *promoter**. The porcine promoter sequence (225 bp) (U70240.1) was aligned with the proximal-most 245 bp of human *APOE *sequence (AF261279). The similarity is 69.1%. Positions of the "TATA" box and the -155SNP site are indicated.

We aligned porcine and human *APOE *promoter sequences. The homology of the proximal promoter regions between human *APOE *from -236 to +10 and porcine *APOE *from -265 to -12 was of greater similarity (69.1%) than those of -266 or more upstream of porcine *APOE*, and a TATA box was found in the two sequences (Figure 1), which suggested functional importance of these fragments across the two species. There is a functional mutation -219T>G in human *APOE *[12], and the -155T>A of porcine *APOE *changes the potential transcription factor binding sites (additional file 1). Thus -155T>A mutation was further studied.

### Effects of -155 mutation on APOE mRNA levels

**Figure 2:**
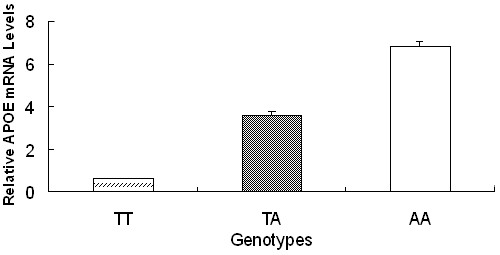
**The T>A transition at position -155 in the porcine *APOE *promoter region increases mRNA levels in Dahuabai pigs**. Data are expressed as means ± S.E.. The mRNA levels of different genotypes differ *P *< 0.01.

To determine whether -155T>A was associated with differential *APOE *expression *in vivo*, hepatic mRNA levels of adult Dahuabai pigs were analyzed with qRT-PCR. The results revealed that mRNA levels of -155AA animals were approximately 11 times higher than those of -155TT animals (Figure 2).

### The -155SNP effects on *APOE *transcription

**Figure 3:**
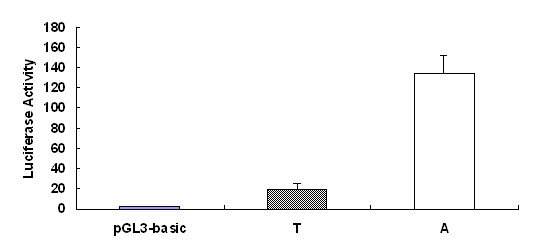
**Functional analysis of porcine *APOE *promoter alleles containing -155T or A**. Data are expressed as means ± S.E.. The activity of the -155A promoter is significantly (*P *< 0.01) higher than that of -155T.

We used the porcine *APOE *promoter region spanning -268 to -11 to assemble fusion constructs of the *APOE *promoter and a firefly luciferase reporter. As shown in Figure 3, the promoter with -155A allele was significantly better at driving luciferase expression, and reporter levels were 6.7-time higher than that with -155T.

### Impact of the -155T>A mutation on DNA-protein interactions

**Figure 4:**
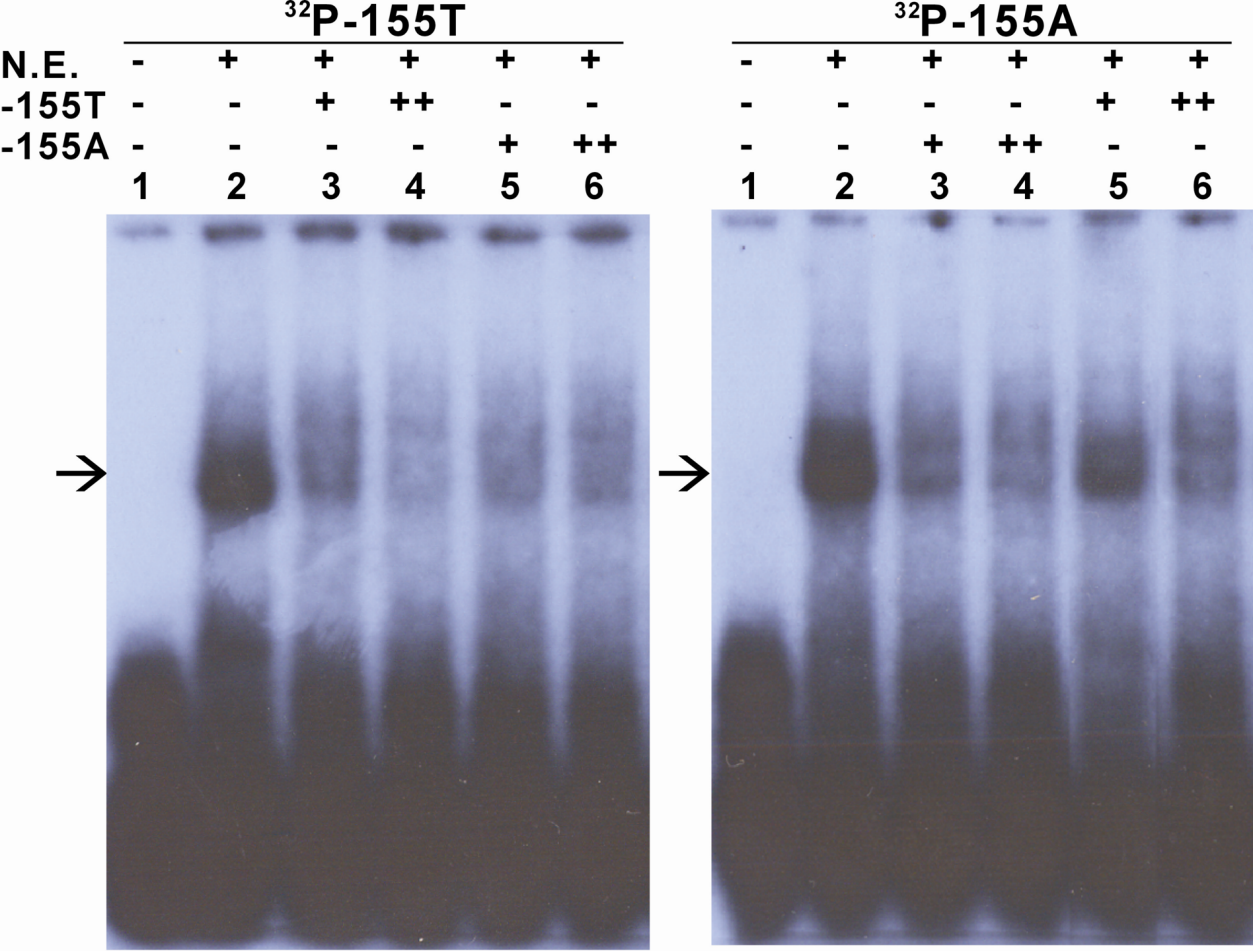
**The T>A transition at position -155 of the porcine *APOE *gene increases DNA-binding affinity of nuclear protein(s) in porcine fetal fibroblasts**. Electrophoretic mobility shift assays were carried out as described in Materials and Methods. Protein-DNA complexes were separated on 5% PAGE and exposed to X-ray film. In competition experiments (lanes 3-6), Nuclear extracts were preincubated in the absence (-) or 10-fold excess (+) or 50-fold excess (++) of unlablled competitor before addition of the ^32^P-labelled probe. Binding complexes identified with the -155T probe and -155A probe were indicated with arrows.

EMSAs were performed to understand whether the differential promoter activities were related to changes in protein binding. As shown in Figure 4, incubation of nuclear extract with the probe corresponding to the -155T or -155A allele predominantly formed a specific binding complex (lane 2 of Figure 4A and 4B, arrowed). The binding complex of -155T probe with transcriptional factor(s) was largely abolished by co-incubation with an excess of either unlabelled -155T or -155A allele probes (lanes 3-6 of Figure 4A). By contrast, the complex formed by incubation of nuclear extract with the -155A probe was largely eliminated by co-incubation with unlabelled -155A competitor (lanes 3, 4 of Figure 4B) or unlabelled 50-fold excess -155T probe (lane 6 of Figure 4B), whereas a conspicuous complex formation was observed in the presence of unlabelled 10-fold excess -155T competitor (lanes 5 of Figure 4B). The results indicated that -155A allele can greatly enhance the binding affinity of transcriptional factor(s).

## Discussion

This study identified a functional SNP in the 5' regulatory region of porcine *APOE*. The behaviours of the allelic forms of the reporter gene expression, real-time PCR and EMSA in our study strongly suggested that -155T>A allelic differences of the *APOE *transcription may be a consequence of differential binding ability of transcriptional factors or differential transcriptional factors bound to a wild type or a mutation type present in cells.

To distinguish potentially functional SNPs from nonfunctional SNPs, we focused on *cis*-acting elements based on the suggestion of Knight [28] and used a predictive program http://www.genomatix.de[27]. The results indicated there were different transcription factors binding the *cis*-acting elements surrounding the -155SNP (additional file 1). The -155T contains a CCCTCCC motif that is known as the SP1 binding site [29], and SP1 has been implicated for its function in regulating AD-associated genes [30]. Meanwhile, the mutation at porcine *APOE *-155 site still had SP1 binding ability.

The existence of multiple transcription factors binding the -155 region was further reinforced by incubation of a -155T/A DNA probe with nuclear extracts from porcine fetal fibroblasts forming DNA-protein complexes as demonstrated by EMSA. Importantly, competition EMSA using excess unlabelled probes revealed a difference in the binding affinities of promoter alleles: excess -155A DNA cold probe effectively abolished complex formation, whereas 10-fold excess -155T DNA cold probe failed to prevent labelled -155A probe-protein complex formation. From the above results, we could consider that the -155A promoter had a significantly higher affinity for binding factors than that for the -155T promoter. Interestingly, for human APOE -491A>T mutation, the -491A displayed an increased affinity for human hepatic nuclear proteins [14], while the -491A showed an opposite trend for rat pheochromocytoma and human SK-N-SH neuroblastoma nuclear proteins [15].

It was demonstrated, in transfected cells, that the cloned promoter containing the -155A variant was significantly stronger than that of the corresponding -155T variant at driving luciferase expression (6.7-fold higher expression, *P *< 0.01). The cloned promoter in this research did not contain either of the other two identified polymorphic sites (-440 and +501). The difference in transcription efficiency was confirmed *in vivo*, and *APOE *mRNA levels were 11 times higher in liver tissue from -155AA homozygous pigs than that in the tissue from -155TT homozygotes (*P *< 0.01). In the report of Maloney et al. [15], human -491A>T variant interacted with -219G>T because both mutations altered the variety and binding affinity of transcription factors. However, the -401SNP and +501SNP in porcine *APOE *were not expected to change the transcription factors, which implied that there may be no interactive effect between porcine *APOE *-155SNP and the other two SNPs.

All these data suggest that polymorphism at SNP-155 modulates transcription of the porcine *APOE *gene by affecting the ability of the *APOE *promoter to bind to *trans*-acting factors. It is notable that the -155 T>A substitution alters the CCCTCCC sequence to CCCACCC. The CACCC box is a well-described *cis*-acting transcription element that can serve as a binding site for widely distributed transcription-activating factors that act collaboratively with other regulatory proteins [31]. There are a larger family of zinc finger transcription factors bound to CACCC box [32]. Mutation of this box is thought to change binding and transactivation by transcription factors and therefore to decrease transcription levels [33-36].

Our results suggest that the -155A allele is associated with increased levels of *APOE *transcription *in vivo*. It seems probable that the -155A variant can elevate levels of apoE protein. We also report that the frequency of the -155AA genotypes is significantly under-represented, compared to -155TT and -155TA, in each of the four different pig populations analyzed. Although the explanation for this depletion is unknown, there are several considerations as follows.

Firstly, it was previously reported that apoE enhanced cell lipid homeostasis [17,37] and could reduce the risk of heart disease [16,38]. In humans, the concentration of apoE in plasma or brain varies according to *APOE *genotype in the order E2 > E3 > E4 [37], and the E4 allele is an established risk factor for AD. The -491A allele of human *APOE *is also a risk factor for AD, but this function is associated with higher levels of *APOE *transcription versus the -491T allele, and these effects are exerted independently of E4 [14]. In the experiments reported here we found that the -155A allele was associated with significantly increased transcription of porcine *APOE *both in transfected cells and hepatic tissue. Higher apoE concentration lead to increased cytotoxicity [39], thus -155A allele may be harmful to porcine survival.

Secondly, some recessive genes that terminate fetal development are known to be located on swine chromosome *SSC6 *between *SW855 *and *SW122 *[40], a region that includes the porcine *APOE *gene [22,40]. The affected gene(s) in the homozygous state can prevent embryo development after 9 days post-coitus when the spherical embryo grows to the filamentous form, leading to embryonic death due to implantation failure [40]. This suggests that under-representation of -155AA could be a consequence of linkage disequilibrium between porcine *APOE *and other genes in the vicinity.

## Conclusions

We have identified a functional SNP in the regulatory region of the porcine *APOE *gene that was associated with altered interactions with DNA-binding factors, marked differences in the activity of the *APOE *promoter and levels of *APOE *mRNA *in vivo*.

## Methods

### Screening for porcine APOE 5' regulatory SNPs

All animal procedures were performed according to protocols approved by the Biological Studies Animal Care and Use Committee of Guangdong Province, China. Ear tissues of Landrace, Yorkshire, Chinese indigenous breed Lantang and Dahuabai pigs were used for genomic DNA isolation according to Sambrook and Russell [41]. Primers (Table 2) for three overlapping segments were designed with Primer Primer 5.0 according to porcine *APOE *sequence (GenBank: U70240.1). The PCR products were sequenced and genotyped.

**Table 2:** Primers for SNP detection in the porcine *APOE *5'-regulatory region

Primers	Sequences	Annealing temperature	PCR product length
P1	Upstream: 5'-TCGAGAGGGAGTGAGGGTTAAAA-3'	59°C	538 bp
	Downstream: 5'-AGGAGCAGGACCAGAAGG-3'		
P2	Upstream: 5'-GGCCTTCTGGTCCTGCTCCT-3'	61°C	455 bp
	Downstream: 5'-AACTCCTGCCTCGGTGGTGG-3'		
P3	Upstream: 5'-CACCGAGGCAGGAGTTGG-3'	60°C	728 bp
	Downstream: 5'-CTTCTGGCTTGCGATTGG-3'		

### Real-time quantitative PCR

Total RNA was extracted from the hepatic tissue of adult Dahuabai pig (-155TT, -155TA, -155AA, 6 animals of each of the three genotypes) with TRIzol reagent (Invitrogen). QRT-PCR analysis was performed using the ABI 7500 system (Applied Biosystems) (primers: 5'-CGCAGGATGCCGGACAGA-3' and 5'-CCTCCTGCACTTGGTCAGACA-3'). The gene expression levels were qualified relative to the expression of *β-actin *by the comparative C_T _method [42].

### Plasmid constructions

The DNA segment spanning the -268 to -11 region of porcine *APOE *and containing either the -155T or -155A variants was amplified with the primers 5'- CACGCGTAGTGGCATGGAGAAA-3' and 5'- ACTCGAGACTCCTCCAGAACT -3', thereby creating the restriction sites of *Mlu*I and *Xho*I (underlined). Following restriction enzyme digestion the PCR products were inserted to vector pGL3-basic (Promega). The integrity of cloned sequences was confirmed by sequencing.

### Transient transfection assays

Porcine fetal fibroblast cell line was established as described previously [43]. Cells (1.5 × 10^6 ^per well) were seeded into 96-well plates and grown to 80-90% confluence, and transfected with *APOE *promoter/firefly luciferase reporter plasmids by *Lipofectamine *2000 transfection reagent (Invitrogen). To control for transfection efficiency, cells were co-transfected with 2 ng of *Renilla *luciferase reporter plasmid (Promega). After 48 h of transfection, cells were lysed and assayed for promoter activity using the dual luciferase reporter assay system. Firefly luciferase activity was expressed relative to *Renilla *luciferase activity.

### Electrophoretic mobility shift assays

Nuclear proteins were extracted from porcine fetal fibroblast cells cultured at third passage (Nuclear Extract Kit, Active Motif). The sequences used for EMSAs were 5'-CCCCTCCCCC(**T/A)**CCCTGCTGTGTG-3' surrounding the -155 site. The oligonucleotides were 5'-labeled with [γ-^32^P]-ATP using a Megalabel DNA 5'-labelling Kit according to the specifications of the manufacturer (Takara). The 5'-labelled probes used in subsequent experiments were double stranded.

The binding mixture included 0.5 μg poly (dI-dC) (Amersham Biosciences), 2 μl of 5 × gel-shift binding buffer, 2 μg of nuclear extract. The mixture was maintained at room temperature for 10 min, 2 μl of radiolabelled oligonucleotide probe was added, and incubation was kept for a further 20 min at room temperature in the presence or absence of a 10- to 50-fold molar excess of unlabelled probes. DNA-protein complexes were fractionated by electrophoresis on 5% non-denaturating polyacrylamide gels.

### Data analysis

For continuous data, comparisons of two means employed independent *t *tests, *q *values were calculated for comparisons of three means. Genotype frequencies were analyzed with probit models [44].

## Abbreviations

APOE: apoliprotein E gene; apoE: apoliprotein E; EMSA: electrophoretic mobility shift assays; SNP: single nucleotide polymorphism; AD: Alzheimer's disease; qRT-PCR: quantitative real-time polymerase chain reaction.

## Authors' contributions

SL conceived and designed the study, performed the EMSAs, designed PCR primers and wrote the paper. HZ designed the study, performed the qRT-PCR, and genotyped the animals, analyzed the data and wrote the paper. PG cultured porcine fetal fibroblast cells, constructed the plasmids and performed transient transfection assays. ZC extracted DNA and RNA, and performed animal genotyping. CW extracted DNA and RNA, and performed qRT-PCR experiments. JL designed the study, supervised the experiments and wrote the paper. All authors read and approved the final manuscript.

## Supplementary Material

Additional file 1 **Transcriptional factors surrounding -155SNP predicted by MatInspector**. Click here for file
