# Supplementary material for: A functional mutation at position -155 in porcine APOE promoter affects gene expression
Source: BMC Genet. 2011 May 9;12:40. doi: 10.1186/1471-2156-12-40 (PMC3098798; doi:10.1186/1471-2156-12-40)
Supplement: Additional file 1 — Transcriptional factors surrounding -155SNP predicted by MatInspector. [file 1471-2156-12-40-S1.DOC]

Transcriptional factors surrounding -155SNP predicted by MatInspector

| -155T | | | | |  | -155A | | | | |
| --- | --- | --- | --- | --- | --- | --- | --- | --- | --- | --- |
| Identical predicted transcriptional factors and binding sites | | | | | | | | | | |
| Factor | Strand | Matrix  Sim. | Core  Sim. | Sequence |  | Factor | Strand | Matrix  Sim. | Core  Sim. | Sequence^*^ |
| PRDM5 | - | 0.714 | 0.96 | ggg**a**gggggAGGGgaggggaggggagggg |  | PRDM5 | - | 0.71 | 0.96 | ggg**t**gggggAGGGgaggggaggggagggg |
| PLAG1 | - | 0.941 | 1 | G**A**GGgggaggggaggggaggg |  | PLAG1 | - | 0.887 | 0.833 | G**T**GGgggaggggaggggaggg |
| KKLF | - | 0.979 | 1 | **a**gggggaGGGGagggga |  | KKLF | - | 0.979 | 1 | **t**gggggaGGGGagggga |
| CKROX | - | 0.958 | 1 | g**a**ggGGGAggggagggg |  | CKROX | - | 0.961 | 1 | g**t**ggGGGAggggagggg |
| SP1 | - | 0.911 | 0.807 | **a**ggGGGAggggaggg |  | SP1 | - | 0.911 | 0.807 | **t**ggGGGAggggaggg |
| ZBP89 | + | 0.981 | 1 | cccctcccctCCCCc**t**ccctgct |  | ZBP89 | + | 0.986 | 1 | cccctcccctCCCCc**a**ccctgct |
| WT1 | - | 0.943 | 0.837 | caggg**A**GGGggagggga |  | WT1 | - | 0.998 | 1 | caggg**T**GGGggagggga |
| ZNF219 | + | 0.937 | 1 | ctcccctCCCCc**t**ccctgctgtg |  | ZNF219 | + | 0.939 | 1 | ctcccctCCCCc**a**ccctgctgtg |
| CTCF | - | 0.847 | 1 | tcacacagcaggg**a**GGGGgaggggagg |  | CTCF | - | 0.834 | 1 | tcacacagcaggg**t**GGGGgaggggagg |
|  | |  | Different predicted transcriptional factors or binding sites | | | | |  |  | |
| Factor | Strand |  |  | Sequence |  | Factor | Strand |  |  | Sequence |
| GAGA | - | 0.783 | 0.75 | agcagGG**A**Gggggaggggaggggag |  | BKLF | - | 0.974 | 1 | gcaGGG**T**gggggagggg |
| KLF6 | - | 0.945 | 1 | ggg**a**GGGGgaggggagg |  | CTCF | - | 0.804 | 1 | gg**t**gggggaggggaGGGGaggggaggg |
| PURα | - | 0.989 | 1 | gg**A**GGGggagggg |  |  |  |  |  |  |
| KKLF | - | 0.942 | 1 | caggg**a**gGGGGagggga |  |  |  |  |  |  |
| CKROX | - | 0.901 | 1 | agcaGGG**A**gggggaggg |  |  |  |  |  |  |
| MAZ | - | 0.903 | 1 | caggG**A**GGgggag |  |  |  |  |  |  |

Note: ^*^The -155 T or -155A base is marked with boxes. Capital letters represent core sequences.
